# SLC4A10 mutation causes a neurological disorder associated with impaired GABAergic transmission

**DOI:** 10.1093/brain/awad235

**Published:** 2023-07-17

**Authors:** James Fasham, Antje K Huebner, Lutz Liebmann, Reham Khalaf-Nazzal, Reza Maroofian, Nderim Kryeziu, Saskia B Wortmann, Joseph S Leslie, Nishanka Ubeyratna, Grazia M S Mancini, Marjon van Slegtenhorst, Martina Wilke, Tobias B Haack, Hanan E Shamseldin, Joseph G Gleeson, Mohamed Almuhaizea, Imad Dweikat, Bassam Abu-Libdeh, Muhannad Daana, Maha S Zaki, Matthew N Wakeling, Lucy McGavin, Peter D Turnpenny, Fowzan S Alkuraya, Henry Houlden, Peter Schlattmann, Kai Kaila, Andrew H Crosby, Emma L Baple, Christian A Hübner

**Affiliations:** RILD Wellcome Wolfson Centre, University of Exeter Medical School, Royal Devon University Healthcare NHS Foundation Trust, Exeter EX2 5DW, UK; Peninsula Clinical Genetics Service, Royal Devon University Healthcare NHS Foundation Trust, Exeter EX2 5DW, UK; Institute of Human Genetics, Jena University Hospital, Friedrich Schiller Universität, 07747 Jena, Germany; Institute of Human Genetics, Jena University Hospital, Friedrich Schiller Universität, 07747 Jena, Germany; Department of Biomedical Sciences, Faculty of Medicine, Arab American University of Palestine, Jenin, P227, Palestine; Molecular and Clinical Sciences Institute, St. George’s University of London, London SW17 0RE, UK; Institute of Human Genetics, Jena University Hospital, Friedrich Schiller Universität, 07747 Jena, Germany; University Children’s Hospital, Salzburger Landeskliniken (SALK) and Paracelsus Medical University (PMU), 5020 Salzburg, Austria; Amalia Children’s Hospital, Radboudumc, 6525 GA Nijmegen, The Netherlands; Institute of Human Genetics, Technische Universität München, 80333 Munich, Germany; RILD Wellcome Wolfson Centre, University of Exeter Medical School, Royal Devon University Healthcare NHS Foundation Trust, Exeter EX2 5DW, UK; RILD Wellcome Wolfson Centre, University of Exeter Medical School, Royal Devon University Healthcare NHS Foundation Trust, Exeter EX2 5DW, UK; Department of Clinical Genetics, Erasmus Medical Center, 3015 GD Rotterdam, The Netherlands; Department of Clinical Genetics, Erasmus Medical Center, 3015 GD Rotterdam, The Netherlands; Department of Clinical Genetics, Erasmus Medical Center, 3015 GD Rotterdam, The Netherlands; Institute of Medical Genetics and Applied Genomics, University of Tuebingen, 72076 Tübingen, Germany; Department of Translational Genomics, Center for Genomic Medicine, King Faisal Specialist Hospital and Research Center, Riyadh 11564, Saudi Arabia; Rady Children’s Institute for Genomic Medicine, San Diego, CA 92123, USA; Department of Neurosciences, University of California, San Diego, La Jolla, CA 92093, USA; Department of Neuroscience, King Faisal Specialist Hospital and Research Center, Riyadh 11564, Saudi Arabia; Department of Biomedical Sciences, Faculty of Medicine, Arab American University of Palestine, Jenin, P227, Palestine; Department of Pediatrics and Genetics, Makassed Hospital and Al-Quds University, East Jerusalem, 95908, Palestine; Department of Pediatrics, Arab Women’s Union Hospital, Nablus, P400, Palestine; Clinical Genetics Department, Human Genetics and Genome Research Institute, National Research Centre, Dokki, Cairo 12622, Egypt; RILD Wellcome Wolfson Centre, University of Exeter Medical School, Royal Devon University Healthcare NHS Foundation Trust, Exeter EX2 5DW, UK; Department of Radiology, Derriford Hospital, Plymouth PL6 8DH, UK; RILD Wellcome Wolfson Centre, University of Exeter Medical School, Royal Devon University Healthcare NHS Foundation Trust, Exeter EX2 5DW, UK; Peninsula Clinical Genetics Service, Royal Devon University Healthcare NHS Foundation Trust, Exeter EX2 5DW, UK; Department of Translational Genomics, Center for Genomic Medicine, King Faisal Specialist Hospital and Research Center, Riyadh 11564, Saudi Arabia; Molecular and Clinical Sciences Institute, St. George’s University of London, London SW17 0RE, UK; Institute for Medical Statistics, Computer Science and Data Science, Jena University Hospital, 07747 Jena, Germany; Molecular and Integrative Biosciences, University of Helsinki, 00014 Helsinki, Finland; Neuroscience Center, Helsinki Institute of Life Science, University of Helsinki, 00014 Helsinki, Finland; RILD Wellcome Wolfson Centre, University of Exeter Medical School, Royal Devon University Healthcare NHS Foundation Trust, Exeter EX2 5DW, UK; RILD Wellcome Wolfson Centre, University of Exeter Medical School, Royal Devon University Healthcare NHS Foundation Trust, Exeter EX2 5DW, UK; Peninsula Clinical Genetics Service, Royal Devon University Healthcare NHS Foundation Trust, Exeter EX2 5DW, UK; Institute of Human Genetics, Jena University Hospital, Friedrich Schiller Universität, 07747 Jena, Germany; Center for Rare Diseases, Jena University Hospital, Friedrich Schiller Universität, 07747 Jena, Germany

**Keywords:** acid-base, gamma aminobutyric acid, NBCN2, NCBE, intellectual disability

## Abstract

SLC4A10 is a plasma-membrane bound transporter that utilizes the Na^+^ gradient to drive cellular HCO_3_^−^ uptake, thus mediating acid extrusion. In the mammalian brain, *SLC4A10* is expressed in principal neurons and interneurons, as well as in epithelial cells of the choroid plexus, the organ regulating the production of CSF.

Using next generation sequencing on samples from five unrelated families encompassing nine affected individuals, we show that biallelic *SLC4A10* loss-of-function variants cause a clinically recognizable neurodevelopmental disorder in humans. The cardinal clinical features of the condition include hypotonia in infancy, delayed psychomotor development across all domains and intellectual impairment. Affected individuals commonly display traits associated with autistic spectrum disorder including anxiety, hyperactivity and stereotyped movements. In two cases isolated episodes of seizures were reported in the first few years of life, and a further affected child displayed bitemporal epileptogenic discharges on EEG without overt clinical seizures. While occipitofrontal circumference was reported to be normal at birth, progressive postnatal microcephaly evolved in 7 out of 10 affected individuals. Neuroradiological features included a relative preservation of brain volume compared to occipitofrontal circumference, characteristic narrow sometimes ‘slit-like’ lateral ventricles and corpus callosum abnormalities.

*Slc4a10* ^−/−^ mice, deficient for SLC4A10, also display small lateral brain ventricles and mild behavioural abnormalities including delayed habituation and alterations in the two-object novel object recognition task. Collapsed brain ventricles in both *Slc4a10*^−/−^ mice and affected individuals suggest an important role of SLC4A10 in the production of the CSF. However, it is notable that despite diverse roles of the CSF in the developing and adult brain, the cortex of *Slc4a10*^−/−^ mice appears grossly intact.

Co-staining with synaptic markers revealed that in neurons, SLC4A10 localizes to inhibitory, but not excitatory, presynapses. These findings are supported by our functional studies, which show the release of the inhibitory neurotransmitter GABA is compromised in *Slc4a10*^−/−^ mice, while the release of the excitatory neurotransmitter glutamate is preserved. Manipulation of intracellular pH partially rescues GABA release.

Together our studies define a novel neurodevelopmental disorder associated with biallelic pathogenic variants in *SLC4A10* and highlight the importance of further analyses of the consequences of SLC4A10 loss-of-function for brain development, synaptic transmission and network properties.

## Teaser

Fasham *et al.* show that biallelic loss-of-function variants in *SLC4A10* cause a neurodevelopmental disorder with learning difficulties, seizures and characteristic brain imaging features. Based on findings *in vitro* and *in vivo*, they propose that dysfunction of GABAergic transmission contributes to the disorder.

## Introduction

A large variety of molecules involved in neuronal signalling, including ligand- and voltage-gated channels, show a remarkable sensitivity to changes in the intracellular and extracellular pH.^1–4^ As a rule, the excitability of neuronal networks is enhanced by alkalosis and suppressed by acidosis,^5–7^ which suggests a fundamental evolutionary role for pH as a neuromodulator during physiological and pathophysiological conditions. Numerous studies have provided evidence for mechanisms that control pH dynamics and actions in microdomains within^8–13^ and outside^12^ brain cells based on the heterogeneous spatial patterns of expression of both pH-sensitive and pH-regulatory proteins, including plasmalemmal Na^+^/H^+^ exchangers,^14^ HCO_3_^−^ transporters^15,16^ as well as intra- and extracellular carbonic anhydrase isoforms.^4^

In mammals, members of the SLC4^15^ and SLC26^16^ gene families have been identified as bicarbonate (HCO_3_^−^) transporters, many of which are associated with monogenic human diseases including distal renal tubular acidosis, haemolytic anaemia, corneal dystrophy, glaucoma and cataracts,^15^ as well as chondrodysplasia, chloride diarrhoea, and hearing loss.^17^ The SLC4 family includes Na^+^-independent Cl^−^/HCO_3_^−^ exchangers, electrogenic and electroneutral Na^+^-HCO_3_^−^ cotransporters and Na^+^-driven Cl^−^/HCO_3_^−^ exchangers that mediate HCO_3_^−^ transport across the plasma membrane^15^ (Supplementary Table 1). SLC4A10 utilizes the transmembrane gradient of Na^+^ to drive cellular net uptake of HCO_3_^−^, and thus mediates acid extrusion. Both cytoplasmic and membrane-bound carbonic anhydrases are involved in the supply of bicarbonate and may thus increase transport rates.^18^ To which extent this is relevant for SLC4A10-mediated transport is yet unclear. Some controversy exists as to whether it acts as an electroneutral Na^+^-HCO_3_^−^ cotransporter (NBCn2), or a Na^+^-coupled Cl^−^/HCO_3_^−^ exchanger (NCBE) under physiological conditions.^19,20^ The expression of *SLC4A10* is predominantly neuronal, but it is also expressed in choroid plexus epithelia^21,22^ and in inner ear fibrocytes.^23^ Mice deficient for SLC4A10 show a reduced brain ventricle size suggesting a role in transepithelial electrolyte transport and production of CSF.^22^ Although neuronal excitability was enhanced *in vitro*,^24^ the experimental seizure threshold was paradoxically increased *in vivo*^22^ and spontaneous seizures were not observed.

In humans, heterozygous genomic deletions comprising all or part of *SLC4A10* have been linked with autistic spectrum disorder, with additional features such as impaired motor and language skills or epilepsy.^25–28^ The causal relevance of these genomic alterations is, however, unclear, as the interpretation of these findings is complicated by contiguous gene deletion. Here, we provide clinical, genetic, functional and mouse-model evidence to determine that autosomal recessive SLC4A10 loss-of-function results in intellectual disability with striking radiological abnormalities of the lateral ventricles, closely mirroring findings in *Slc4a10* knockout (KO) mice. As SLC4A10 localizes to inhibitory presynapses and its disruption compromises γ-aminobutyric acid (GABA) release, we propose that alterations of the GABAergic system contribute to the pathomechanistic basis of this neurodevelopmental disorder.

## Materials and methods

### Clinical studies

All families were recruited with written informed consent according to international guidelines, including the Declaration of Helsinki, and regional ethical approvals (Palestinian Health Research Council PHRC/HC/518/19, Technische Universität München, Muenchen Exome Seq.: 5360/12 S, KFSHRC RAC # 2121053, Erasmus MC METC 2012-387, IRB protocol number 150765). Affected individuals were examined and investigated by local clinicians according to routine clinical standards relevant to their clinical presentation.

### Genetic studies

**Figure 1:**
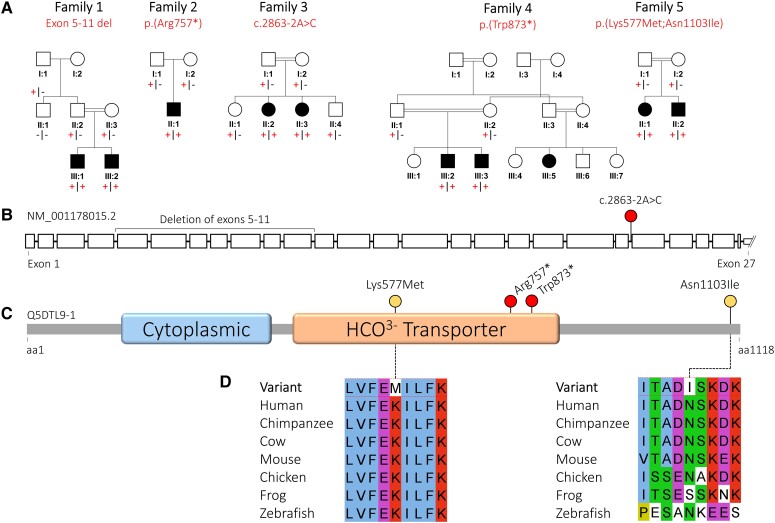
**Family pedigrees and biallelic *SLC4A10* variants.** (**A**) Simplified family pedigrees for individuals affected with *SLC4A10*-related neurodevelopmental disorder, showing autosomal recessive segregation of *SLC4A10* variants. Co-segregation confirmed in other family members as indicated, in each case the plus symbol indicating variant allele and the minus sign indicating wild-type allele. (**B**) Simplified *SLC4A10* exon structure (NM_001178015.2) showing location of the multi-exon deletion identified in Individuals III:1 and III:2 (Family 1) and the splicing variant (c.2863-2A>C). Only a part of the large, non-coding, UTR exon 27 is shown. (**C**) Simplified SLC4A10 protein structure (Q5DTL9-1) showing location of missense (yellow) and predicted loss-of-function (red) variants in relation to the predicted domain architecture (Pfam domains: https://www.ebi.ac.UK/interpro/) of SLC4A10. Cytoplasmic = band 3 cytoplasmic domain (PF07565); HCO_3_^−^ transporter = HCO_3_^−^ transporter family (PF00955). (**D**) Multi-species alignments of SLC4A10, showing each of the missense variants identified in this study. aa = amino acids.

DNA and RNA were extracted from blood/buccal samples using standard techniques. In all five families, whole genome sequencing (WGS) (Family 1) or whole exome sequencing (WES) (Families 2–5) was undertaken to identify the cause of disease. Family pedigrees illustrating the relationships of affected and unaffected individuals in this study are shown in Fig. 1. Unless otherwise specified, genomic variants were filtered based on call quality, predicted consequence, segregation with the disease phenotype and allele frequency in population databases (variants with a frequency of >0.1% and/or present in >1 homozygous individual in gnomAD v2.1.1, v3.1.1 or in-house databases were excluded). Homozygous, compound heterozygous, X chromosome and *de novo* (when trio sequencing undertaken) variants present in exons or within ±6 nucleotides in the intron that remained after filtering, were assessed for clinical correlation with the affected individual(s) phenotype.

In Family 1, WGS was performed (BGI) on DNA from two affected individuals (Family 1, Subjects III:1 and III:2). Reads were aligned (BWA-MEM v0.7.15), mate-pairs fixed, and duplicates removed (Picard v2.7.1), InDel realignment/base quality recalibration (GATK v3.7.0), single-nucleotide variant (SNV)/InDel detection (GATK HaplotypeCaller), annotation (Alamut v1.11), and read depth ascertained using an in-house pipeline. This conforms to GATK best practices. Copy number variants (CNVs) were detected using SavvyCNV.^29^

In Family 2, DNA from the proband (Subject II:1) and both unaffected parents underwent trio WES (Illumina) at Technical University München/Helmholtz Institute Neuherberg using the SureSelect50Mbv5 capture, as previously described.^30,31^ In Family 3, trio WES of a single affected individual (Subject II:2) and both parents was undertaken as previously described (ID: 17-4393).^32^ In Family 4, WES was performed on the two affected brothers at University of California San Diego (UCSD) using methods previously described,^33^ with recessive variants within regions of homozygosity prioritized given the consanguineous nature of the family. In Family 5, trio WES was performed on both affected siblings and their two unaffected parents (four individuals in total) using Agilent SureSelect Target Enrichment Clinical Research Exome V2 (Agilent Technologies). Sequencing (paired-end 150 bp) was performed by the Illumina HiSeq 4000 platform (Illumina, outsourced). Data were demultiplexed by Illumina Software CASAVA. Reads are mapped to the genome (build hg19/GRCh37) with the program BWA (http://bio-bwa.sourceforge.net/). Variants were detected with the Genome Analysis Toolkit (http://www.broadinstitute.org/gatk/). Variants were filtered with the Cartagenia/Alissa Interpret software package (Agilent technologies) on quality (read depth ≥10), frequency in databases (≥1% in 200 alleles in dbSNP, ESP6500, the 1000 Genome project or the ExAC database) and location (within an exon or first/last 10 bp of introns).

In Family 1, unique primers for droplet digital polymerase chain reaction (ddPCR) (QX200 AutoDG Droplet Digital PCR System, Bio-Rad) were designed for confirmation and co-segregation of the exon 5–11 *SLC4A10* deletion (NM_001178015.2:c.417_1341del) (Supplementary Fig. 1). In addition to two primers within the deletion (within exons 5 and 10), probes included an exon 5′ to the deletion (exon 4), an exon 3′ to the deletion (exon 11) and a housekeeping gene control (*RPP30*). Primer sequences are provided (Supplementary Fig. 2).

In Family 3, reverse transcription PCR (RT-PCR), using standard techniques, was undertaken on lymphoblast cell lines derived from affected and controls individuals to confirm the transcriptional outcome of the *SLC4A10* NM_001178015.2:c.2863-2A>C variant. RNA was extracted using RNeasy kit (QIAGEN Cat. No. 74104) as per the manufacturer’s protocol. cDNA was generated from 1 µg of RNA via iScript Select cDNA Synthesis kit (Bio-Rad). Primers that cover exons 19–24 of *SLC4A10* transcript were used for RT-PCR to check for difference in splicing between affected and control lymphoblast.

In Families 2–4, dideoxy sequencing confirmation and co-segregation of single nucleotide *SLC4A10* variants was performed using standard techniques.

### Cellular studies

#### Cloning

The human *SLC4A10* cDNA was cloned by PCR from a human cDNA library and subcloned into the pBI-CMV4 vector (Clontech Cat. No. PT4443-5), a mammalian bidirectional expression vector designed to constitutively express a protein of interest and DsRed2, a human codon-optimized variant of the *Discosoma* species red fluorescent protein. Disease-associated single nucleotide variants were inserted by site-directed mutagenesis and verified by sequencing.

#### Cell culture

N2a cells were cultured at 37°C with Dulbecco’s Eagle’s minimum essential medium (DMEM) (Gibco Cat. No. 31966-021) supplemented with fetal bovine serum to a final concentration of 10% and 1% penicillin/streptomycin (Gibco Cat. No. 15070063). N2a cells were transfected with Lipofectamine 3000 (Invitrogen Cat. No. L3000008) according to the manufacturer’s instructions.

For staining, cells were fixed with 4% paraformaldehyde (PFA) in PBS for 10 min and subsequently washed. Cells were stained with wheat germ agglutinin (WGA) coupled to Biotin (Biozol Cat. No. B-1025) at a dilution of 1:500 and a polyclonal rabbit anti-SLC4A10 antibody (1:500)^22^ at 4°C overnight. The secondary antibodies we used were a Streptavidin-Alexa Fluor 488 conjugate (1:1000, Invitrogen Cat. No. 32354) and an Alexa Fluor 546-coupled goat anti-rabbit antibody (1:1000, Invitrogen). Transfection rates varied between 20% and 40% with no obvious effect of the genotype of the transfected construct. Analysis was done with a confocal microscope in the Airyscan mode (LSM 880, Zeiss). The plasma membrane region (PMR) was determined as the WGA-labelled cell rim.

#### Intracellular pH recordings

Forty-eight hours after transfection, the intracellular pH (pH_i_) was measured using the ratiometric 2′,7′-bis(2-carboxyethyl)-5(6)-carboxyfluorescein (BCECF, Molecular Probes) fluorescent dye. Cells were washed with bicarbonate-buffered solution containing (in mM): 99 NaCl, 20 Na-gluconate, 5 KCl, 1 MgSO_4_, 1.5 CaCl_2_, 25 NaHCO_3_ and 10 glucose. Coverslips were transferred to a heated perfusion chamber (Chamlide EC; Live Cell Instruments, 37°C), which was mounted at an Axio Observer.Z1 microscope (Zeiss). An image was acquired for the RFP channel to identify transfected cells. Thereafter, BCECF-AM was added to a final concentration of 4 µM and incubated for 10 min. The cells were superfused with bicarbonate-buffered solution at a linear flow rate of 2.5 ml/s. Emitted light of 510–535 nm was recorded after alternating excitation at 495 and 440 nm every 10 s and captured through a 10× objective with a CCD-camera (AxioCam MRm; Zeiss). The steady state pH_i_ was recorded for 5 min. Then 5 µM EIPA was added to the perfusion buffer to block Na^+^/H^+^ exchange activity and the pH recorded for another 5 min. The superfusion was then switched to bicarbonate-buffered solution containing 5 µM EIPA and 20 mM sodium propionate instead of 20 mM Na^+^-gluconate for 5 min. After the propionate pulse cells were superfused again with the former used bicarbonate-buffered solution supplemented with 5 µM EIPA. The cytoplasmic pH recovery was recorded during superfusion with 20 mM sodium propionate containing bicarbonate-buffered solution. For each coverslip more than 12 neighbouring transfected and non-transfected cells were analysed and data from different coverslips were averaged. At the end of each experiment, a calibration was done with buffers between pH 6.5 and 7.5 (in mM: 135 KCl, 20 *N*-methyl-D-glucamine, 4 MgSO_4_, 10 glucose, 30 HEPES, 10 µM nigericin). A linear regression was calculated from the multipoint calibration curve, and F_495_/F_440_ ratio was converted into pH_i_ values. Data regarding initial pH, amplitude of acidosis, recovery and pHi amplitude overshoot were obtained, with recovery from acidification being the primary outcome.

### Mouse studies

The generation of *Slc4a10*^−/−^ mice from a 129SvJ embryonic stem cell line was described previously.^22^ All experiments were conducted according to the German Law on the Protection of Animals and the corresponding European Communities Council Directive of 24 November 1986 (86/609/EEC) and were approved by the Thüringer Landesamt für Lebensmittelsicherheit und Verbraucherschutz (Thuringia State Office for Food Safety and Consumer Protection) under the registration number 02-001/13. Mice were group-housed on a 12-h light-dark cycle and fed with food and water *ad libitum*. If not indicated otherwise, the experiments started when the animals were 3 to 4 months old and weighed 25–35 g. Tests were performed during the light phase between 10:00 a.m. and 5:00 p.m.

The two-object novel object recognition (NOR) task was used to evaluate recognition memory in rodents in 12-month-old wild-type and knockout mice of both sexes. During habituation, the animals were allowed to explore an open field arena on 2 days with 1 day interval in between. One week after habituation, the animals were again exposed to the familiar arena but with two identical glass bottles with a blue cap placed at an equal distance. Four hours later, the mice were placed in the arena, after one glass bottle was replaced by a tower of yellow and green Lego® bricks of the same height. Mice were recorded with a CCD camera (Panasonic) for 10 min. The time spent exploring each object, the number of visits and the exploring time per visit were analysed off-line with Microsoft Windows Movie Maker. The primary outcome was the difference score (time exploring novel object − time exploring familiar object) with the discrimination ratio (time exploring novel object/total time spent with both objects) also calculated.

#### Histology and immunohistochemistry

Haematoxylin and eosin (HE) staining followed standard protocols (Carl Roth). For immunofluorescence, brains of 2 to 3-month-old wild-type mice were prepared and fixed as described previously.^8^ Free-floating cryosections (50 µm) were stained with a polyclonal rabbit anti-NeuN antibody (1:1000, Abcam, ab104225) or polyclonal rabbit anti-SLC4A10 antibody.^22^ For co-staining, the following primary antibodies were used: polyclonal guinea pig anti-vesicular GABA transporter (VGAT, 1:250, Synaptic Systems), polyclonal guinea pig anti-vesicular glutamate transporter 1 (VGLUT1, 1:500, Synaptic Systems). Alexa Fluor 488- and 546-coupled goat anti-rabbit and goat anti-guinea pig antibodies were used as secondary antibodies (1:1000, Invitrogen). Cell nuclei were stained by 4,6-diamidino-2-phenylindole (DAPI) (1 µg/ml, Sigma-Aldrich). Analysis was performed with a confocal microscope in the Airyscan mode (LSM 880, Zeiss). To quantify the degree of co-localization, planes were selected with an optimized signal-to-noise ratio using the range indicator and adjusting it to the linear, non-saturated range. Images were taken randomly from the hippocampal CA1 region (stratum radiatum or stratum pyramidale 225 × 225 µm) of four different wild-type brains. The relative area of co-localization was analysed according to the Costes method.^34^ Pearson coefficient was calculated as a measure of co-localization for each image using the co-localization module of ZEN (Release 4.8.2).

#### Slice preparation for electrophysiological recordings

Mice between 2 and 3 months of age were decapitated and the brain was removed from the skull and chilled (at ∼4°C) in artificial CSF (aCSF) containing (in mmol/l): 120 NaCl, 3 KCl, 5 MgSO_4_, 1.25 NaH_2_PO_4_, 0.2 CaCl_2_, 10 D-glucose, and 25 NaHCO_3_, gassed with 95% O_2_–5% CO_2_. Horizontal brain slices (350 μm) including the hippocampus were prepared with a vibroslicer (Leica VT 1200S). Slices were stored at room temperature for at least 1 h before use in recording aCSF containing (in mmol/l): 120 NaCl, 3 KCl, 1.3 MgSO_4_, 1.25 NaH_2_PO_4_, 2.5 CaCl_2_, 10 D-glucose, and 25 NaHCO_3_, gassed with 95% O_2_–5% CO_2_, as described previously.^35^

#### Patch clamp recordings

One slice at a time was placed in a recording chamber mounted on an upright microscope (Axio Examiner.A1; Zeiss) with differential interference contrast, 40× water-immersion objective, and 10× ocular to identify cells. The slices were continuously perfused with aCSF (flow rate 2–3 ml/min, room temperature, pH 7.3) consisting of (in mM): 120 NaCl, 3 KCl, 1.3 MgCl_2_, 2.5 CaCl_2_, 25 NaHCO_3_, 1.25 KH_2_PO_4_, and 10 D-glucose.

For whole-cell recordings patch pipettes with an impedance of ∼3–4 MΩ were pulled from borosilicate glass (OD 1.5 mm; Science Products) with a micropipette puller (P-97, Sutter Instrument) and filled with intracellular solutions for miniature excitatory postsynaptic currents (mEPSC) or miniature inhibitory postsynaptic currents (mIPSC) recordings, respectively.

Pyramidal neurons of the CA1 and CA3 were selected for recording if they displayed a pyramidal-shaped cell body. Patched cells were voltage clamped. Only cells with a resting membrane potential below −55 mV and an access resistance <15 MΩ were included. Therefore, it was not necessary to compensate for the series resistance. Voltages were corrected for liquid junction potentials or series resistance. Signals were recorded using a patch-clamp amplifier (MultiClamp 700B; Axon Instruments). Responses were filtered at 5 kHz and digitized at 20 kHz (Digidata 1440A; Axon Instruments). All data were acquired, stored and analysed on a PC using pClamp 10 (Axon Instruments).

MEPSCs and mIPSCs were recorded at a holding potential of −70 mV for at least 5 min in aCSF. MEPSCs were isolated by adding tetrodotoxin (0.5 μM, Tocris Bioscience) to block action potential-induced glutamate release and bicuculline methiodide (20 μM, Biomol) to block GABA_A_ responses. Dl-APV (30 μM) was added to suppress NMDA currents. The pipette solution contained the following (in mM): 120 CsMeSO_4_, 17.5 CsCl, 10 HEPES, 5 BAPTA, 2 Mg-ATP, 0.5 Na-GTP, 10 QX-314 [*N*-(2,6-dimethylphenylcarbamoylmethyl) triethylammonium bromide], pH 7.3, adjusted with CsOH.

Recordings of mIPSCs were performed using a CsCl-based intracellular solution (in mM): 122 CsCl, 8 NaCl, 0.2 MgCl_2_, 10 HEPES, 2 EGTA, 2 Mg-ATP, 0.5 Na-GTP, 10 QX-314 [*N*-(2,6-dimethylphenylcarbamoylmethyl)triethylammonium bromide], pH adjusted to 7.3 with CsOH. dl-APV (30 μM), CNQX (10 μM) and tetrodotoxin (0.5 μM) were added to the perfusate. Recordings of spontaneous inhibitory postsynaptic currents (sIPSCs) were performed in the absence of tetrodotoxin.

In a subset of mIPSC experiments, 20 mM NaCl was substituted by the weak base trimethylamine chloride (TriMA; Sigma-Aldrich) to raise pH_i_. In another subset of mIPSC experiments, 20 mM NaCl was substituted by the weak acid sodium propionate (Sigma-Aldrich) to lower pH_i_. After a baseline recording of 5 min, the regular aCSF was replaced by aCSF with either TriMA or sodium propionate, and mIPSCs were recorded for further 5 min.

For mIPSC recordings in bicarbonate-free extracellular solution we used (in mM): 130 NaCl, 3 KCl, 1.3 MgSO_4_, 1.25 NaH_2_PO_4_, 2.5 CaCl_2_, 10 D-glucose, 10 HEPES, gassed with O_2_, pH 7.3 with NaOH.

The following parameters of mEPSCs and mIPSCs/sIPSCs were determined: frequency, peak amplitude, time constant of decay (τ_decay_), half-width and electrical charge transfer, with analysis of frequency the primary outcome measure. Data analysis was performed off-line with the detection threshold levels set to 5 pA for mEPSCs and mIPSCs because of the peak-to-peak noise determined under AMPA/NMDA receptor and GABA_A_ receptor blockade.

### Statistical analysis

Data are presented as mean ± standard error of the mean (SEM).

To address potential issues with statistical analysis of the data associated with small sample sizes, the distribution of the test statistics and corresponding *P*-values were obtained using the bootstrap method (1000 replicates were applied as suggested in Dwivedi *et al.*^36^).

Comparison of two statistically independent experimental groups was performed with the two-tailed *t-*test. If data were dependent, the paired *t*-test was used. In experiments that included more than two groups, differences were tested by an *F*-test.

For correlated, replicated data we used a GEE model using normal errors identity link and independent working correlation matrix. Calculations were performed in R and RStudio using the package gee for the GEE model.^37–39^

## Results

### Genetic analysis

We initially investigated the cause of disease in two male Palestinian siblings (aged 7 and 8 years) affected by a syndromic form of severe intellectual disability, behaviours associated with autistic spectrum disorder, slit ventricles and subtle craniofacial dysmorphism (Family 1). The younger child was microcephalic, with an occipitofrontal circumference (OFC) of 3.4 standard deviation scores below the mean (−3.4 SDS), whereas his older brother had an OFC of −2.3 SDS. To define the genetic cause of disease WGS was performed on DNA from both affected children (Family 1, Subjects III:1 and III:2). Filtering of WGS data using standard metrics described above identified a single standout candidate variant, a shared, homozygous out-of-frame deletion of exons 5–11 of *SLC4A10* [Chr2(GRCh38):g.161846109-161895992del; NM_001178015.2:c.417_1341del p.(Trp140Argfs*39)] clearly visible on genome sequencing (Supplementary Fig. 1) and predicted to result in nonsense-mediated decay (NMD) and absence of the SLC4A10 protein. The variant was confirmed using ddPCR as an orthologous method and found to co-segregate as expected for an autosomal recessive disorder (Supplementary Fig. 2).

Through collaborative studies (via GeneMatcher) we then identified seven additional affected individuals from four unrelated families (Fig. 1), in whom WES identified biallelic rare predicted loss-of-function *SLC4A10* variants (Fig. 1, Table 1, Supplementary material, ‘Case reports’ and Supplementary Tables 2 and 3 for family pedigrees, clinical details, comprehensive case reports, *SLC4A10* variants and WGS/WES variant lists, respectively). These individuals (aged 4–17 years) presented with clinical features overlapping those of the Palestinian children. In Family 2, trio WES (Subject II:1) identified a homozygous nonsense variant in exon 18/27 [Chr2(GRCh38):g.161949151C>T; NM_001178015.2:c.2269C>T p.(Arg757*)] also expected to undergo NMD. Family 3 included two sisters with global developmental impairment identified as part of a large-scale study aiming to identify candidate new genetic causes of disease.^32^ Trio WES of DNA from the older sister (Family 3, Subject II:2) identified a homozygous canonical splice site variant Chr2(GRCh38):g.161964133A>C; NM_001178015.2:c.2863-2A>C, also confirmed to be homozygous in her affected sibling. RT-PCR revealed that the variant resulted in partial intron retention and a premature stop codon [r.(2772_2773ins2772+1_2772+175); r.(2773_2781del)]; p.(Gln954_Phe955ins*13) expected to result in NMD (Supplementary Fig. 3). In Family 4, WES performed on DNA from two brothers, (Family 4, Subjects III:2 and III:3) identified a shared homozygous *SLC4A10* nonsense variant in exon 20/27 expected to result in NMD [Chr2(GRCh38):g.161957066G>A NM_001178015.2:c.2619G>A p.(Trp873*)]. In Family 5, WES performed on DNA from two siblings and their parents identified a shared homozygous *SLC4A10* haplotype comprising two missense variants, Chr2(GRCh38):g.161904888A>T NM_001178015.2:c.1730A>T p.(Lys577Met) and Chr2(GRCh38):g.161976840A>T NM_001178015.2:c.3308A>T; p.(Asn1103Ile), hereafter referred to as p.(Lys577Met;Asn1103Ile). The p.(Lys577Met) variant affects an invariantly conserved residue within a helical transmembrane domain and is predicted deleterious by *in silico* tools PolyPhen2 and SIFT with a high REVEL score (0.873), whereas p.(Asn1103Ile) affects a highly conserved residue but is predicted deleterious only by SIFT and benign by PolyPhen with a low REVEL score (0.239) (Supplementary Table 2). All the *SLC4A10* variants identified in this study are absent from the Genome Aggregation Database (gnomAD v2.1.1 and v3.1.2); furthermore, there are no homozygous loss-of-function variants in canonical *SLC4A10* transcripts listed in publicly accessible genomic databases.

**Table 1:** Clinical findings in individuals with biallelic *SLC4A10* variants

Individual	Family 1 III:1	Family 1 III:2	Family 2 II:1	Family 3 II:2	Family 3 II:3	Family 4 III:2	Family 4 III:3	Family 4 III:5	Family 5 II:1	Family 5 II:2
NM_001178015.2	Homozygous deletion of exons 5–11	Homozygous deletion of exons 5–11	Homozygous p.(Arg757*)	Homozygous c.2863-2A>C p.(Gln954_Phe955ins*13)	Homozygous c.2863-2A>C p.(Gln954_Phe955ins*13)	Homozygous p.(Trp873*)	Homozygous p.(Trp873*)	Unknown	Homozygous p.(Lys577Met;Asn1103Ile)	Homozygous p.(Lys577Met;Asn1103Ile)
Sex, age	M, 8 y 10 m	M, 7 y 8 m	M, 4 y 8 m	F, 12 y	F, 4 y	M, 10 y	M, 6 y 3 m	F, 17 y 5 m	F, 11 y	M, 6 y
Ethnicity	Palestinian	Palestinian	European	Arab Saudi	Arab Saudi	Egyptian	Egyptian	Egyptian	Turkish	Turkish
Birth OFC	NK	NK	NK	Normal	NK	34.2 (−0.8)	35 (−0.2)	33(−1.3)	NK	NK
OFC, cm (SDS)	50.5 (−2.3)	48.5 (−3.4)	50 (−1.7)	47.5 (−4.5)	44.5 (−5.5)	45.5 (−5.6)	46.6 (−4.3)	48 (−5.4)	51.6 (−1.9)	46.7 (−4.2)
Height, cm (SDS)	NK	NK	101.5 (−1.3)	125 (−0.4)	100 (−0.4)	123 (−2.5)	104 (−2.7)	150 (−2.2)	136 (−1.2)	111 (−1.0)
Weight, kg (SDS)	NK	NK	10.7 (−4.9)	19.8 (−1.8)	10.9 (−3.5)	23 (−2.3)	16 (−2.5)	45 (−1.8)	30.4 (−0.9)	17 (−1.7)
Feeding difficulties	NK	NK	Yes	NK	Yes at birth	No	No	Yes	Yes	Yes
**Neuro/development**										
Intellectual disability	Yes, severe	Yes, severe	Yes, severe	Yes, severe	Yes, severe	Yes, severe	Yes, severe	Yes, severe	Yes, moderate	Yes, severe
Gross motor	Walked >2 y	Walked 5 y	Rolling	Crawling	Not rolling	Walked 6 y	Walked 6 y	Walked 7 y	Walked 2 y	Walked 3 y
Speech	Non-verbal	Non-verbal	Babbles	Babbles	Sounds	Non-verbal	Non-verbal	Non-verbal	Dysarthria	Non-verbal
Hearing loss	No	No	No	NK	NK	No	No	No	No	No
Anxiety	Yes	Yes	No	Yes	No	Yes	Yes	Yes	No	Yes
Stereotypies	No	Yes	No	Yes	No	Yes	Yes	Yes	No	No
Hyperactivity	Yes	Yes	No	No	No	Yes	Yes	Yes	No	No
Seizures	Yes	Yes?	No	Yes GTCS	No	Abn. EEG	No	No	No	No
Central tone	↓	↓	↓	↓	↓	↓	↓	↓	↓	↓
Peripheral tone	↑	↑	↓	↑	↓	↓	↓	↓	↓	↑
Tendon reflexes	+++	+++	++	+++ and clonus	NK	++	++	++	++	+++
**MRI brain**										
Slit lateral ventricles	Yes	Yes	Yes	NK	Yes	NK	NK	NK	Yes	Yes
Dysmorphic CC	Yes	Yes	Yes	NK	Yes	NK	NK	NK	No	Yes
Fornix/SP	Yes	Yes	Yes/No	NK	Yes	NK	NK	NK	No	Yes
**Other findings**										
Other		Inverted nipples	Coxa vara anteverta, DDH	Ankle contractures	Craniosynostosis, ankle contractures				Accessory nipple	

Yes = feature is present; No = feature is absent; +++ = exaggerated or brisk; ++ = normal; downwards arrow = decreased; upwards arrow = increased; Abn. EEG = abnormal EEG; CC = corpus callosum; DDH = developmental dysplasia of the hip; F = female; Fornix/SP = distorted configuration of fornix/septum pellucidum; GTCS = generalized tonic-clonic seizures; M = male; m = months; NK = not known; OFC = occipitofrontal circumference; SDS = standard deviation scores from the mean; y = years.

### Clinical features of *SLC4A10*-related neurodevelopmental disorder

All 10 affected individuals presented with hypotonia in infancy, with resultant significant feeding difficulties in four of the individuals. Psychomotor development was delayed in all individuals across all domains and intellectual impairment was typically severe. Affected individuals were non-verbal, with one exception; although 7/10 children were ambulatory, walking was delayed in these children until between 2–7 years of age. There was no evidence of developmental regression and while hearing loss was noted in a *Slc4a10*^−/−^ mouse model,^23^ it was not reported in any of the affected patients in this study. Seizures were reported in three individuals, but in two cases these were isolated episodes occurring in the first few years of life. In addition, an affected child from Family 4 displayed bitemporal epileptogenic discharges on EEG at age 5 years in the absence of overt clinical seizures, with spontaneous resolution thereafter.

Behavioural abnormalities were very commonly present and included features associated with autistic spectrum disorder such as anxiety and stereotyped movements (hand flapping, head nodding), hyperactivity and in some cases aggressive episodes. OFC was reported to be normal at birth, but recent measurements were below average in all cases (−1.7 SDS to −5.6 SDS) with 7 of 10 affected individuals meeting the criteria for microcephaly (<−3 SDS). Affected individuals were below average weight for their age, with height relatively preserved.

**Figure 2:**
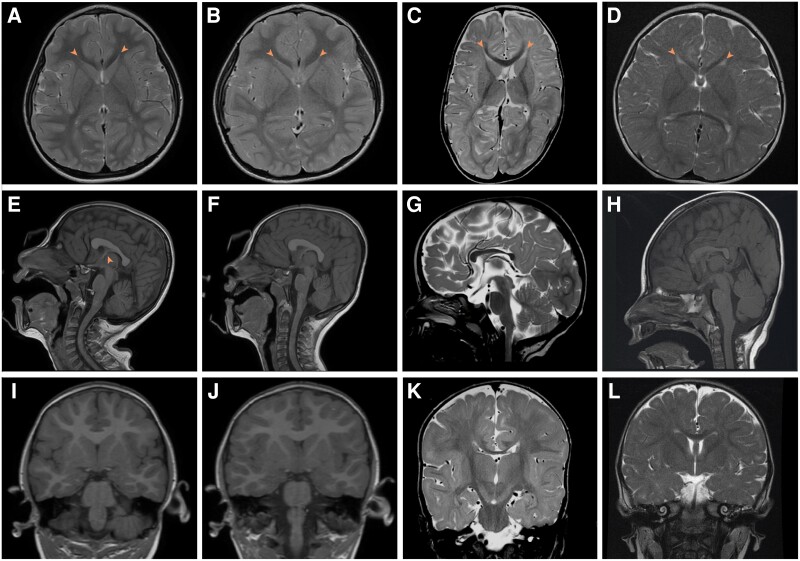
**Neuroimaging from affected individuals with biallelic SLC4A10 variants.** (**A**, **E** and **I**) Family 1, Subject III:1. T_2_-weighted axial (**A**), T_1_-weighted sagittal (**E**) and T_1_-weighted coronal (**I**) MRI images of the patient at age 5 years. (**B**, **F**, **J**) Family 1, Subject III:2. T_2_-weighted axial (**B**), T_1_-weighted sagittal (**F**) and T_1_-weighted coronal (**J**) MRI images of the patient at age 4 years. (**C**, **G** and **K**) Family 2, Subject II:1. T_2_-weighted axial (**C**), T_2_-weighted sagittal (**G**) and T_2_-weighted coronal (**K**) MRI images of the patient at10 months of age. (**D**, **H** and **L**) Family 3, Subject II:3. T_2_-weighted axial (**D**), T_1_-weighted sagittal (**H**) and T_2_-weighted coronal (**L**) MRI images at 1 year 2 months of age. In all cases lateral ventricles are small (**A**–**D**, arrowhead) with normal fourth ventricle (**E**–**H**), posterior fossa and external CSF spaces. In Family 1, the corpus callosum is dysmorphic, appearing thickened and flattened (**E** and **F**). This is associated with an unusual configuration of the fornix and septum pellucidum especially in Family 1, Subject III:1 (**E**, arrowhead). In Families 2 and 3 it is hypoplastic (**G** and **H**). Myelination is complete or adequate for age in all cases. Normal MRI brain images for comparison are available at https://www.imaios.com/en/e-Anatomy/Brain/Brain-MRI-in-axial-slices (adult) and https://radiopaedia.org/cases/normal-mri-head-3-years-old-1 (3-year-old child).

MRI neuroimaging findings were striking and consistent. Neuroradiological features of the *SLC4A10*-related neurodevelopmental disorder included microcephaly, with a relative preservation of brain volume compared to OFC and narrow, sometimes slit-like lateral ventricles similar to those seen in the *Slc4a10*^−/−^ mouse model (Supplementary Fig. 4), even in those cases with less well preserved cerebral volume. The corpus callosum was either normal, or dysmorphic (slightly thickened and blunted, flattened in a cranio-caudal direction and with sharply descending fornix) (Fig. 2 and Supplementary Fig. 5). This is likely to be as a result of the small lateral ventricles displacing the fornix and septum pellucidum. There was an absence of cortical malformations and myelination was appropriate for age.

### Recovery from acidification is delayed in cells expressing disease-associated SLC4A10 variants

We previously showed that acid extrusion is compromised in hippocampal neurons in acute brain slices from knockout mice.^22^ Here, we sought to provide insight into the functional consequences of the *SLC4A10* missense variants using cellular studies. We first cloned wild-type and variant *SLC4A10* cDNAs into the mammalian expression vector pBI-CMV4. Two days post-transfection into the fast-growing mouse neuroblastoma cell line N2a, cells were fixed with 4% PFA and stained with an antibody directed against an N-terminal epitope of SLC4A10 as described previously and with the lectin WGA to label glycan structures associated with the plasma membrane.^22,40^ As expected, cells transfected with the wild-type *SLC4A10* construct displayed a predominant labelling at the plasma membrane, whereas the SLC4A10 p.(Lys577Met;Asn1103Ile) variant protein showed a predominant intracellular localization (Supplementary Fig. 6A). The quantification of signal intensities for the interior of cells (not including the WGA labelled surface) as compared to the plasma membrane region (the WGA labelled surface) allowed us to calculate the ratio between cell surface and intracellular intensities, which was significantly increased for the SLC4A10 p.(Lys577Met;Asn1103Ile) variant protein (Supplementary Fig. 6B).

While this outcome alone may explain the pathogenic mechanism of the SLC4A10 p.(Lys577Met;Asn1103Ile) variant, we also assessed the impact on transport activity by BCECF fluorescence imaging in transfected N2a cells in bicarbonate-buffered salt solution with or without 5 µM EIPA to block Na^+^/H^+^ exchange. Representative single cell traces are shown in Supplementary Fig. 7A. Compared with untransfected cells, steady state pH_i_ was slightly more alkaline in cells transfected with the *SLC4A10* wild-type construct (Supplementary Fig. 7B). A shift in pH_i_ remained for both the p.(Lys577Met) and the p.(Asn1103Ile) variant proteins but was present to a lesser extent for the combined p.(Lys577Met;Asn1103Ile) variant (Supplementary Fig. 7C). Bath application of 20 mM sodium propionate for 5 min induced an acid shift, the amplitude of which did not differ between wild-type and mutant constructs (Supplementary Fig. 7D). pH_i_ recovery during the propionate exposure was significantly faster for the wild-type construct compared to untransfected cells (transfected cells 163.1 ± 22.9%, untransfected cells 100.0 ± 18.7%, *n* = 7/7, bootstrap paired *t*-test *P* = 0.001, Supplementary Fig. 7E). For p.(Lys577Met), p.(Asn1103Ile) and p.(Lys577Met;Asn1103Ile) the alkaline overshoot after propionate removal (which provides a quantification of net removal of acid during the propionate exposure) was significantly smaller compared to wild-type [bootstrap *F*-test *P* < 0.001, *post hoc* tests: wild-type (WT) 287.5 ± 18.2%, p.(Lys577Met) 169.2 ± 14.0%, *n* = 7/10, bootstrap *t*-test *P* < 0.001, WT 287.5 ± 18.2%, p.(Asn1103Ile) 231.8 ± 24.9%, *n* = 7/9, bootstrap *t*-test *P* < 0.05, WT 287.5 ± 18.2%, p.(Lys577Met;Asn1103Ile) 139.6 ± 18.6%, *n* = 7/8, bootstrap *t*-test *P* < 0.001] (Supplementary Fig. 7F). Taken together, we conclude that acid extrusion is significantly diminished in cells expressing SLC4A10 p.(Lys577Met) and p.(Asn1103Ile) variants alone and p.(Lys577Met;Asn1103Ile) *in cis*.

### *Slc4a10* ^−/−^ mice show behavioural abnormalities and display grossly intact cortical structure

**Figure 3:**
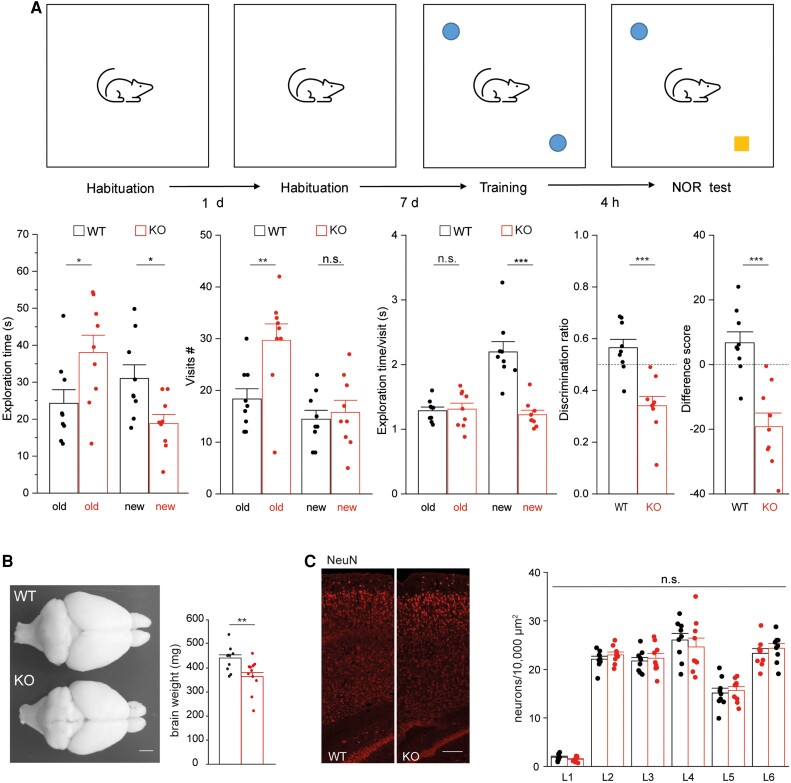
***Slc4a10* ^−/−^ mice show behavioural abnormalities in the two-object novel object recognition task and display grossly intact cortical architecture**. (**A**) The recognition of the novel object is altered in knockout (KO) mice. *Top*: Illustration of the two-object novel object recognition (NOR) test. *Bottom*: During the NOR test the exploration time, the number of visits for the old and the new, and the duration of these visits were quantified. A difference score (time exploring novel object − time exploring familiar object) and the discrimination ratio (time exploring the novel versus the familiar object) was calculated (nine mice per genotype, bootstrap *t*-test; *P < 0.05; **P < 0.01, ***P < 0.001). A negative value for the difference score or a value smaller 0.5 (dashed lines) for the discrimination ratio suggest that exploration is altered. (**B**) Top view of dissected brains from 12-month-old *Slc4a10* wild-type (WT) and KO mouse. The weight of perfused and fixed brains of KO mice was smaller compared to WT (*n* = 5 mice per genotype; bootstrap *t*-test; **P < 0.01). Scale bar = 2 mm. (**C**) The gross architecture of the somatosensory cortex appeared intact in *Slc4a10* KO mice. Sagittal brain sections from 2-month-old *Slc4a10* WT and KO mice were stained for the pan neuronal marker NeuN and neurons counted layer wise (*n* = 3 mice per genotype; GEE model using normal errors identity link and independent working correlation matrix). Scale bar = 75 µm. Quantitative data are presented as mean + standard error of the mean (SEM). n.s. = not significant.

The identification of biallelic *SLC4A10* variants in affected individuals with cognitive impairment and behaviours associated with autistic spectrum disorder prompted us to reanalyse the behaviour of *Slc4a10*^−/−^ mice. In our previous paper, we reported that motor functions including activity, locomotion and motor coordination were not altered in *Slc4a10* knockout mice.^22^ Here, we used the two-object NOR task to assess recognition memory, which is based on the spontaneous tendency of rodents to spend more time exploring a novel object than a familiar one.^41^ Interestingly, *Slc4a10* knockout mice clearly displayed a marked avoidance of the novel object (Fig. 3A).

As structural brain abnormalities were present in some of the affected patients, we also reanalysed the brain structure of *Slc4a10*^−/−^ mice. Overall, the brain was found to be smaller and the weight reduced in knockout mice compared to wild-type animals (Fig. 3B). As previously reported,^22^ we also noted smaller brain ventricles in *Slc4a10*^−/−^ mice, while the corpus callosum appeared intact (Supplementary Fig. 4).

To test whether *Slc4a10*^−/−^ mice display abnormalities in cortex organization, we also counted neurons labelled for the pan-neuronal marker NeuN (RBFOX3)^42^ in sagittal sections of the motor and the somatosensory cortex of 2-month-old adult mice. Overall, the number of neurons per layer did not differ between genotypes (Fig. 3C), suggesting an absence of any gross cortical layering defect in *Slc4a10*^−/−^ mice.

### SLC4A10 modulates GABAergic but not glutamatergic transmission

**Figure 4:**
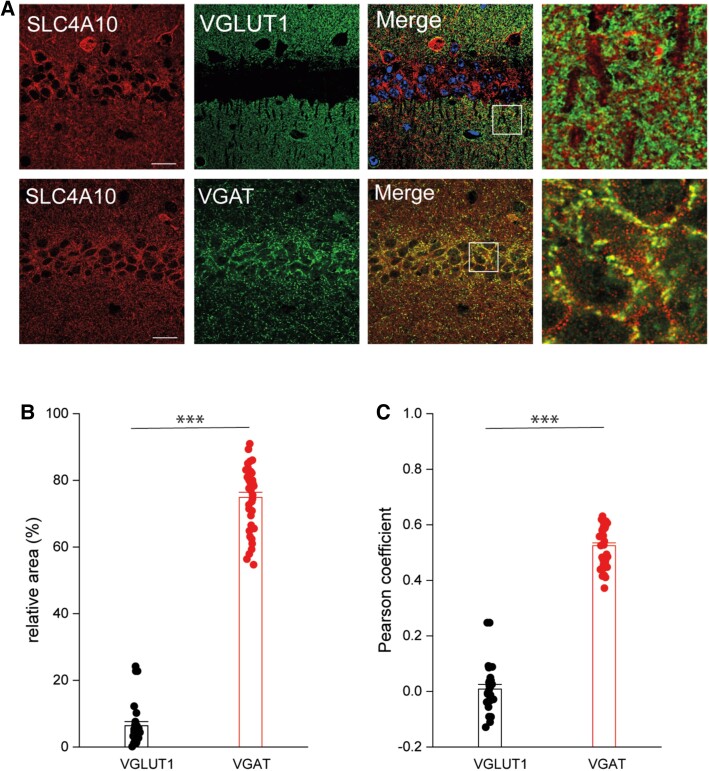
**Localization of SLC4A10 to GABAergic presynapses.** *Slc4a10* wild-type (WT) mouse brain sections. Scale bars = 20 µm, enhanced view of merged marker images also shown (boxed areas). (**A**) VGLUT1, a marker of excitatory presynaptic terminals, rarely co-localizes with SLC4A10 in the CA1 region of the hippocampus (green: SLC4A10, red: VGLUT1). (**B**) SLC4A10 and VGAT, a marker for GABAergic presynapses, co-localize in the CA1 region of the hippocampus (green: SLC4A10, red: VGAT). (**C**) Quantitative analysis of co-localization of SLC4A10 with either VGLUT1 or VGAT and calculation of Pearson correlation coefficients between these data in the CA1 region of the hippocampus (VGLUT1 *n* = 28 and VGAT *n* = 39 images each, bootstrap *t*-test; ***P < 0.001).

To gain further insight regarding the role of SLC4A10 in neuronal functions, we co-stained mouse brain sections for SLC4A10 and either VGLUT1, a presynaptic marker of excitatory synapses, or VGAT (Fig. 4A), a presynaptic marker of inhibitory synapses. We have previously published control staining on knockout tissues.^22^ Whereas the relative area of co-localization was 6.4 ± 0.5% (*n* = 28) for VGLUT1, it was 74.9 ± 1.3% (*n* = 39) for VGAT (Fig. 4B). Pearson correlation coefficients (CC) between SLC4A10 and either VGLUT1 or VGAT signals after the Costes method^34^ are in agreement with a predominant localization of SLC4A10 with GABAergic but not glutamatergic presynapses [Fig. 4B, CC VGLUT1/SLC4A10 (0.03) versus CC VGAT/SLC4A10 (0.5), 28 and 39 images each].

**Figure 5:**
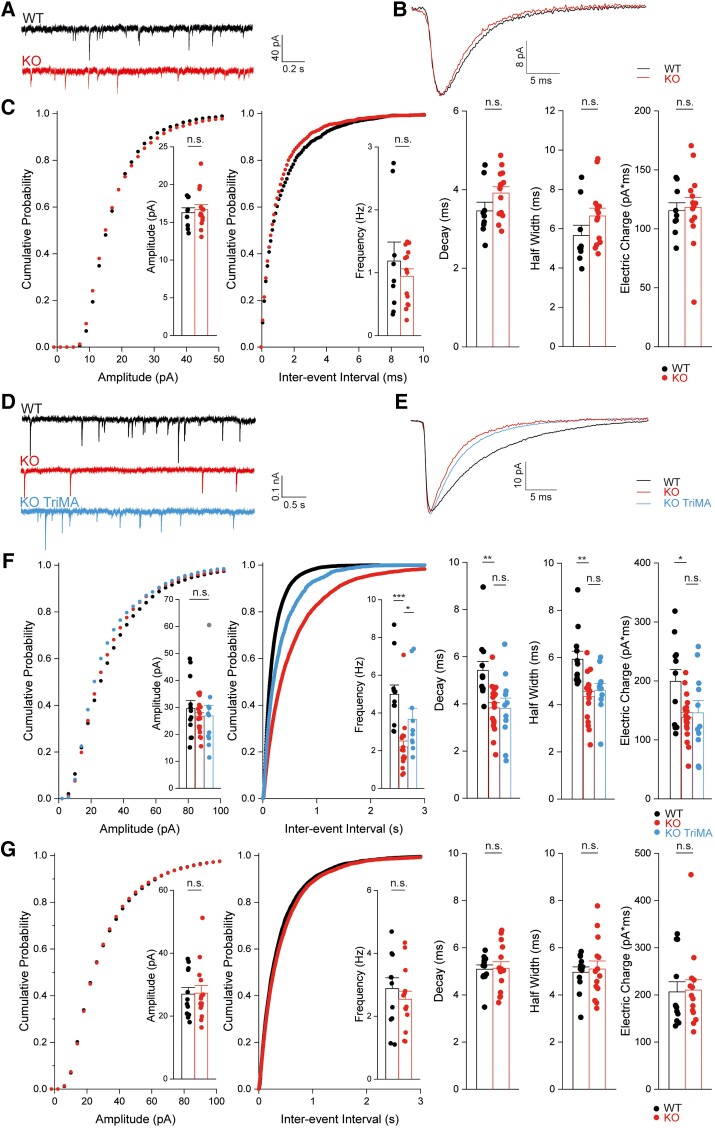
**SLC4A10 acts on presynaptic pH_i_ to promote GABA release in CA1 pyramidal neurons**. Glutamatergic transmission is not impaired in CA1 neurons of *Slc4a10*^−**/**−^ mice (**A**–**C**). (**A**) Representative miniature excitatory postsynaptic current (mEPSC) recordings of pyramidal neurons from *Slc4a10* wild-type (WT) and knockout (KO) mice. (**B**) Averaged mEPSCs show that the kinetics of mEPSCs are not affected by disruption of *Slc4a10*. (**C**) Cumulative plots and bar charts of different mEPSC properties. No significant differences were detected in mEPSC frequency, amplitude or kinetics (*n* = 9/14; bootstrap *t*-test). n.s. = not significant. (**D**–**G**) The miniature inhibitory postsynaptic currents (mIPSC) frequency is diminished in *Slc4a10*^−/−^ mice in the presence of bicarbonate. (**D**) Representative recordings of ongoing mIPSC activity in pyramidal neurons from *Slc4a10* WT and KO mice as well of pyramidal neurons from *Slc4a10* KO mice in the presence of 20 mM trimethylamine chloride (TriMA). (**E**) Averaged mIPSC recordings of pyramidal neurons from *Slc4a10* WT and KO mice to illustrate kinetics and amplitude. (**F**) Cumulative plots and bar charts of mIPSC properties (*n* = 12/19/11; bootstrap *F*-test with *post hoc* analysis: *P < 0.05; **P < 0.01; ***P < 0.001; n.s. = not significant). While no differences in the mean amplitudes of mIPSCs were observed, the frequency of mIPSCs was significantly diminished in cells derived from *Slc4a10* KO mice but could be partially rescued by application of TriMA. Diminished τ_decay_ and half-width of averaged mIPSCs in pyramidal neurons from *Slc4a10* KO mice compared with WT in bicarbonate-buffered artificial CSF were not affected by TriMA. (**G**) In HEPES-buffered nominally bicarbonate-free solution mIPSC frequencies and kinetics did not differ between genotypes (*n* = 14/12; bootstrap *t*-test: *P < 0.05; **P < 0.01; ***P < 0.001). Quantitative data are shown as mean + standard error of the mean (SEM).

These data led us to next study whether neurotransmitter release is affected in the CA1 region of the hippocampus *Slc4a10*^−/−^ mice. Frequency, amplitude and kinetics of mEPSCs recorded in the presence of TTX did not differ between genotypes (Fig. 5A–C and Supplementary Table 4) suggesting that glutamate release is not affected by disruption of SLC4A10. In contrast, the frequency of mIPSCs in TTX, either recorded in CA1 (Fig. 5D–F and Supplementary Table 4) or CA3 (Supplementary Fig. 8), were significantly decreased in the presence of HCO_3_^−^. While amplitudes were unaffected, τ_decay_ and consequently the transferred electric charge per event were diminished in slices obtained from *Slc4a10*^−/−^ mice. As there is evidence that spontaneous and evoked neurotransmission are partially segregated at inhibitory synapses,^43^ we also studied sIPSCs, the frequencies and kinetics of which were reduced (Supplementary Fig. 9).

As the disruption of the acid-extruder SLC4A10 is expected to decrease neuronal pH_i_,^22^ we tested whether the mIPSC frequency can be rescued by raising pH_i_. Indeed, 20 mM trimethyl ammonium (TriMA), which raises pH_i_ without affecting pH_o_,^44^ increased the mIPSC frequency in preparations from *Slc4a10*^−/−^ mice (Fig. 5D–F), while the kinetics were not affected. Analogously, lowering pH_i_ by replacing 20 mM NaCl by sodium propionate decreased mIPSC frequency in slices from wild-type mice (Supplementary Fig. 10). The effects of the disruption of *Slc4a10* on frequency and kinetics were eliminated under bicarbonate-free conditions in recordings performed in HEPES-buffered solution, arguing against structural defects or an altered subunit composition of postsynaptic GABA_A_ receptors (Fig. 5G).

Together, these data show that SLC4A10 modulates GABAergic synaptic transmission in a HCO_3_^−^-dependent manner.

## Discussion

Here we present clinical and genetic data from five unrelated families, alongside molecular and neurobiological findings in mice that define biallelic loss-of-function variants in *SLC4A10* as a cause of a severe neurodevelopmental disorder, frequently associated with microcephaly (<−3 SDS) and morphologically abnormal collapsed (slit) lateral ventricles. This slit-like appearance of the lateral ventricles appears to be characteristic of the disorder and mirrors findings in the *Slc4a10*^−/−^ mouse.^22^ SLC4A10 mediates Na^+^-dependent acid extrusion at the basolateral side of choroid plexus epithelial cells.^22^ Thus, collapsed brain ventricles in knockout mice and in patients with *SLC4A10* biallelic loss-of-function alleles suggest that basolateral SLC4A10-dependent Na^+^ uptake plays a key role for the apical Na^+^-coupled secretion of the CSF.^45^

The four truncating *SLC4A10* variants identified are predicted to result in complete molecular loss-of-function [deletion of exons 5-11; p.(Trp140Argfs*39), p.(Arg757*), p.(Trp873*) and c.2863-2A>C; p.(Gln954_Phe955ins*13)]. Consistent with this, affected individuals homozygous for these variants have the most severe neurological outcomes. Additionally, while both the p.(Lys577Met), affecting the transmembrane region (Supplementary Fig. 1^1^), and C-terminal p.(Asn1103Ile) variant proteins were each trafficked to the proximity of the plasma membrane individually, SLC4A10 protein harbouring both p.(Lys577Met;Asn1103Ile) variants *in cis* was largely trapped intracellularly and acid extrusion was shown to be significantly diminished (Supplementary Figs 6A and 7E and F), strongly supportive of pathogenicity.

Previously a *de novo* balanced translocation disrupting *SLC4A10* was identified as a candidate cause of disease in a single individual described to have ‘mental retardation, progressive cognitive decline, and partial complex epilepsy’.^26^ However, a heterozygous *SLC4A10* variant causing a severe monogenic disease is not consistent with the autosomal recessive condition described here, given the unaffected parental/sibling carriers of loss-of-function *SLC4A10* variants, and the many heterozygous loss-of-function gene variants listed in gnomAD. While it remains unclear whether an undetected *SLC4A10* variant may have been present *in trans* with the disrupted *SLC4A10* allele, heterozygous loss of SLC4A10 function due to the translocation event alone appears unlikely to be responsible for the neurological condition affecting this individual.

Our findings are also of note in light of recent genome-wide association studies (GWAS), which identify a highly statistically significant association between *SLC4A10* intronic or *incis* regulatorytranscription-binding region variants and neurological traits including cognitive function, educational attainment, brain and hippocampal volume and psychiatric morbidity (Supplementary Table 5).^46–48^ Taken together with our present findings, these data provide compelling evidence for the importance of *SLC4A10* in normal neurological development and function and suggest a potential role for *SLC4A10* in traits mediated by oligo/polygenic inheritance.

Notably, *Slc4a10*^−/−^ mice show altered object discrimination with avoidance of the novel object thus resembling a mouse model of autistic spectrum disorder.^49^ This prompted us to use our mouse model to further characterize the role of SLC4A10 in brain function. We previously showed that SLC4A10 is broadly expressed in both principal cells and inhibitory interneurons and that its disruption impaired the recovery of neurons from an acid load in the somatodendritic compartment.^22^ Here, we show that SLC4A10 co-localizes with a marker of GABAergic but not glutamatergic presynapses. In agreement with this localization, GABA release was reduced, while glutamate release was not affected. This defect is characterized by a decrease of mIPSC frequency, while mIPSC amplitudes remain unaltered. Intracellular alkalinization with TriMA partially rescued mIPSC frequency in brain slices from *Slc4a10*^−/−^ mice, while intracellular acidification induced a decrease of mIPSC frequency in wild-type mice, which further supports the conclusion that the difference in mIPSC frequency between the two genotypes are pH_i_ dependent. The knockout of a plasma membrane resident Na^+^-coupled anion exchanger such as SLC4A10 might also change the equilibrium potential for Na^+^ (E_Na_) thus coupling E_Na_ to pH. It is conceivable that changes in E_NA_ lead to subtle changes in membrane potential. Such voltage fluctuations can spread along axons and thus modulate the amplitude of axonal action potentials and postsynaptic potentials evoked by these spikes.^50^ However, cellular Na^+^ loading by pH-regulatory mechanisms typically requires blocking the Na-K ATPase.^51^ Moreover, the relative permeability for Na^+^ of a typical neuron at rest is very low compared to K^+^ and Cl^−^ and thus exerts only a minor contribution to the resting membrane potential.^52^ In agreement, the resting membrane potential between wild-type and knockout principal neurons did not differ at steady state, thus excluding a major effect of SLC4A10 on E_Na_ and excitability of principal neurons. A limitation of the current study is that we have not studied the basic electrophysiological properties of interneurons. However, given that the basic properties of principal neurons are largely unaffected by the disruption, it is highly likely that this also applies to interneurons.

In contrast to the decreased mIPSC frequency, mIPSC kinetics were only mildly changed, which can reflect alterations at the postsynaptic site such as changes in the receptor density or the composition of the receptor subunits.^53^ However, the differences between genotypes were abolished under bicarbonate-free conditions which eliminates the activity of Na^+^-dependent HCO_3_^−^ transporters arguing against this possibility. Because GABA_A_ receptor function critically depends on extracellular pH,^4,54,55^ changes in the kinetics rather suggest an increase in the pH of the synaptic cleft. Accordingly, TriMA, which only raises pH_i_, but does not affect extracellular pH,^56^ did not change mIPSC kinetics. Notably, the disruption of the Na^+^/H^+^ exchanger NHE1/SLC9A1, another transporter expressed at inhibitory presynapses, also decreased mIPSC frequency and altered kinetics.^11^

Thus, both SLC4A10 and SLC9A1 seem likely to contribute to the regulation of pH_i_ at GABAergic nerve endings and, notably, biallelic variants in *SLC9A1* have been linked to a syndromic neurological disorder.^57^ Furthermore, control of pH_i_ at glutamatergic presynapses is mediated by the combination of SLC9A1^11^ and SLC4A8,^58^ another Na^+^-dependent HCO_3_^−^ transporter closely related to SLC4A10. Similar to the defect of GABA release upon disruption of SLC4A10, disruption of SLC4A8 affects glutamate release via its effect on presynaptic pH_i_. These data show that changes in pH_i_ may affect the vesicle release machinery in both GABAergic and glutamatergic neurons in numerous ways. Presynaptic Ca^2+^ transients, which trigger synaptic vesicle exocytosis,^59^ may be altered upon disruption of either *Slc4a8*, *Slc4a10* or *Slc9a1*, potentially because both Ca^2+^ influx via voltage-gated Ca^2+^ channels (VDCCs)^2,60,61^ and Ca^2+^ release from intracellular stores^62,63^ are strongly pH-dependent. Alternatively, H^+^ may compete with Ca^2+^ at the binding site of synaptic vesicles, or may alter the function of proteins involved in vesicle release.^64^ Changes in pH_i_ might also affect the loading of GABA into synaptic vesicles, because VGAT operates as a GABA/H^+^ exchanger and critically depends on the H^+^ electrochemical gradient generated by the vacuolar-type H^+^-ATPase.^65,66^ However, the lack of effect of disruption of SLC4A10 on mIPSC amplitudes are evidence against such an effect.^67^

Consistent with a defect of GABA release, we previously reported an increased network excitability in acute brain slices obtained from *Slc4a10* knockout mice as evidenced by compromised paired-pulse facilitation and increased excitatory postsynaptic potential-spike coupling (E-S coupling).^24^ Changes in the production and composition of the CSF of *Slc4a10* knockout mice may have opposite effects on network excitability *in vivo*. Indeed, seizure susceptibility to pentylenetetrazole (PTZ) and hyperthermia-induced hyperventilation with respiratory alkalosis were diminished in *Slc4a10* knockout mice.^22^ Whether patients with *SLC4A10-*related disease are at increased risk of developing seizures is as of yet unclear, and in our study only 2 out of 10 patients had a clear history of epilepsy.

Patients with *SLC4A10* loss-of-function not only suffer from intellectual disability and behavioural abnormalities, but also show microcephaly and characteristic slit-like brain ventricles. Both the strong expression of SLC4A10 in choroid plexus epithelial cells^21,22^ and the collapsed brain ventricles characteristic of this disease, are indicative of a severely reduced production of CSF in patients. Historically the CSF was primarily considered to provide a simple supportive environment for the brain. However, it is now appreciated that the CSF is an integral component of the CNS with dynamic and diverse roles, which commence during early brain development, for example maintaining the stemness of embryonic progenitor cells, which contact the CSF via their apical surface.^68^ Moreover, explants cultured as neurospheres depend on age-matched CSF in order to maintain appropriate progenitor identity, proliferation and neuronal differentiation.^69,70^ While our analyses did not identify gross alterations of the cortical structure in *Slc4a10* knockout mice, additional studies will be required to rule out more subtle structural changes. As CSF components exchange with the interstitial fluid of the brain parenchyma, changes in CSF production and composition may influence periventricular brain structures such as the hippocampus and hypothalamus, but also potentially other brain structures that exchange with the interstitial fluid.^71^ To identify potential consequences on synaptic functions, in the future it would be beneficial to generate and compare phenotypes of mice with a disruption of SLC4A10 either specifically in neurons, or in the choroid plexus.

In summary, we present extensive genetic, clinical, functional and murine datasets that confirm that biallelic *SLC4A10* pathogenic loss-of-function gene variants cause a syndromic neurodevelopmental disorder. Defects of GABAergic function are a recurrent finding in various neurodevelopmental and neuropsychiatric phenotypes such as intellectual disability, autistic spectrum disorder, epilepsy and schizophrenia.^72,73^ Importantly, positive modulation of GABA_A_ receptors by diazepam and GABA_A_ receptor agonists have been shown to improve behavioural and neurophysiological defects in mouse models of fragile X syndrome.^74,75^ Given this, it is tempting to hypothesize that enhancing inhibitory GABAergic transmission could be a possible therapeutic approach for ameliorating some of the neurological symptoms in patients with *SLC4A10*-related neurodevelopmental disorder.

## Supplementary Material

awad235_Supplementary_Data

## Data Availability

Full WGS and WES sequencing data are not available due to reasons of confidentiality; anonymized variant data will be made available on reasonable request. The authors declare that all other data are contained within the manuscript and the Supplemental material. *SLC4A10* variants have been deposited in ClinVar with submission number SUB11166749.
